# Patterns of Diversity and Humoral Immunogenicity for HIV-1 Antisense Protein (ASP)

**DOI:** 10.3390/vaccines12070771

**Published:** 2024-07-13

**Authors:** Diogo Gama Caetano, Paloma Napoleão-Pêgo, Larissa Melo Villela, Fernanda Heloise Côrtes, Sandra Wagner Cardoso, Brenda Hoagland, Beatriz Grinsztejn, Valdilea Gonçalves Veloso, Salvatore Giovanni De-Simone, Monick Lindenmeyer Guimarães

**Affiliations:** 1 AIDS and Molecular Immunology Laboratory, Oswaldo Cruz Institute, Oswaldo Cruz Foundation, Rio de Janeiro 21040-360, Brazilmonicklg@ioc.fiocruz.br (M.L.G.); 2 Epidemiology and Molecular Systematics Laboratory (LEMS), Oswaldo Cruz Institute, Oswaldo Cruz Foundation, Rio de Janeiro 21040-360, Brazil; 3 Instituto Nacional de Infectologia Evandro Chagas, Oswaldo Cruz Foundation, Rio de Janeiro 21040-360, Brazil; 4 Center for Technological Development in Health (CDTS), National Institute of Science and Technology for Innovation in Neglected Population Diseases (INCT-IDPN), Oswaldo Cruz Foundation, Rio de Janeiro 21040-900, Brazil; 5 Program of Post-Graduation on Science and Biotechnology, Department of Molecular and Cellular Biology, Biology Institute, Federal Fluminense University, Niterói 22040-036, Brazil

**Keywords:** HIV-1, ASP, antisense protein, subtypes, humoral response

## Abstract

HIV-1 has an antisense gene overlapping env that encodes the ASP protein. ASP functions are still unknown, but it has been associated with gp120 in the viral envelope and membrane of infected cells, making it a potential target for immune response. Despite this, immune response patterns against ASP are poorly described and can be influenced by the high genetic variability of the env gene. To explore this, we analyzed 100k HIV-1 ASP sequences from the Los Alamos HIV sequence database using phylogenetic, Shannon entropy (Hs), and logo tools to study ASP variability in worldwide and Brazilian sequences from the most prevalent HIV-1 subtypes in Brazil (B, C, and F1). Data obtained in silico guided the design and synthesis of 15-mer overlapping peptides through spot synthesis on cellulose membranes. Peptide arrays were screened to assess IgG and IgM responses in pooled plasma samples from HIV controllers and individuals with acute or recent HIV infection. Excluding regions with low alignment accuracy, several sites with higher variability (Hs > 1.5) were identified among the datasets (25 for worldwide sequences, 20 for Brazilian sequences). Among sites with Hs < 1.5, sequence logos allowed the identification of 23 other sites with subtype-specific signatures. Altogether, amino acid variations with frequencies > 20% in the 48 variable sites identified were included in 92 peptides, divided into 15 sets, representing near full-length ASP. During the immune screening, the strongest responses were observed in three sets, one in the middle and one at the C-terminus of the protein. While some sets presented variations potentially associated with epitope displacement between IgG and IgM targets and subtype-specific signatures appeared to impact the level of response for some peptides, signals of cross-reactivity were observed for some sets despite the presence of B/C/F1 signatures. Our data provides a map of ASP regions preferentially targeted by IgG and IgM responses. Despite B/C/F1 subtype signatures in ASP, the amino acid variation in some areas preferentially targeted by IgM and IgG did not negatively impact the response against regions with higher immunogenicity.

## 1. Introduction

The HIV genome is classically described as composed of nine genes that encode structural, regulatory, and accessory proteins with well-characterized roles in viral biosynthesis [1]. Alternative open reading frames (ORFs) can be identified within other genomic elements, including antisense frames [2,3,4,5]. Although most antisense ORFs are involved in producing non-coding RNAs that participate in gene expression regulatory mechanisms, HIV-1 presents an ORF encoding the antisense protein ASP.

The ASP ORF was first described by Miller et al. in 1988 as a codifying antisense ORF that overlaps the *env* gene and produces a protein with a length of approximately 190 amino acids, an estimated molecular weight of 20 kDa, and a high level of hydrophobicity, suggesting its association with lipid membranes [6]. Moreover, evidence of ASP expression was provided by studies that observed the presence of antisense RNA transcripts [7,8] and the identification of polyadenylation signals and genetic promoters on the 3′ tail [3,8,9,10,11]. A comprehensive molecular study of the ASP gene conducted by Cassana et al. revealed its specificity to group M of HIV-1 and identified a selection pressure favoring the maintenance of the gene in the most prevalent subtypes [12]. Furthermore, two immunolocalization studies have highlighted that ASP functions as a virion component, indicating a role as an accessory protein [13,14]. 

Although ASP’s function is still unknown and a generally low expression level of antisense RNAs in infected cells [8,9,10,15] raises ongoing controversies about the relevance of ASP in HIV pathogenesis, some studies have detected ASP expressed in the viral envelope and the plasmatic membrane of infected cells [13,14,16]. Affram et al. characterized ASP as a protein associated with gp120 in stimulated infected cells from several myeloid and lymphoid HIV-1-infected cell lineages [16], indicating that ASP could be a potential target for the humoral immune response. Despite that, data regarding immunogenicity patterns of the ASP protein are scarce in the literature, with only two studies that aimed to evaluate the presence of a humoral response against ASP [17,18].

Based on this, our goal was to characterize the humoral immunologic response against ASP, identifying primary immunogenic regions within ASP from the most prevalent HIV-1 subtypes present in Brazil while considering the influence of the most common mutations on the level of antibody response.

## 2. Materials and Methods

### 2.1. Dataset Analysis

By April 2021, we searched the Los Alamos HIV Sequence Database (LAHSD) [19] for all nucleotide sequences containing the ASP gene fragment (positions 7373–7942 about the HXB2 reference sequence). The selected sequences were retrieved along with their respective information regarding the isolation country and HIV subtype. The obtained sequences were grouped into two datasets: one dataset containing all ASP sequences from LAHSD (Dataset ASP-ORF Full/*n* = 99,363 sequences) and the second one containing only one sequence per patient (Dataset ASP-ORF PerPatient/*n* = 25,861 sequences).

From each dataset, sequences from the most prevalent HIV-1 subtypes in Brazil (B, C, and F1) were selected based on their previous genetic classification and sorted in different sequence lists according to the geographic information (Brazil or worldwide). A full list containing sequences’s accession number, subtype, country, and sampling year is presented in Supplementary Table S1, and sequence counts by geographic and subtype information are summarized in Supplementary Table S2.

For each sequence list, nucleotide sequences were translated on frame -1 and further aligned using the MAAFT algorithm FFT-NS2 [20] implemented in Geneious v2021.1 software [21]. Based on this alignment, defective sequences with stop codons or frameshift mutations that compromised alignment in at least 70% of the amino acid sequence were removed from sorted sequence lists, followed by realignment of the filtered sequence lists. 

Alignments were further divided into six sections: three sections equivalent to overlapping REV Response elements, gp120 V5, and gp120 V4 regions, which presented hypervariability and a higher frequency of insertions and deletions, and three more conserved sections flanking hypervariable regions. Each ASP region was further realigned using the MAAFT algorithm FFT-NS-i-2x and concatenated with its respective alignments.

For phylogenetic analyses, final alignments were merged with an alignment containing reference sequences for subtypes D, B, C, and F1 using the profile alignment tool from Geneious. These merged alignments were then used to construct maximum likelihood phylogenetic trees using the Fasttree2 algorithm [22] implemented in Geneious software to confirm the HIV classification and evaluate country-related clusterizations. Maximum likelihood trees were further edited and formatted using the iTOL v6 software [23].

First, an ASP alignment for each HIV-1 subtype (worldwide or in Brazil) was obtained for genetic diversity analyses. After that, gaps were manually inserted in the hypervariable alignment regions to equalize the alignment length and overlap the low-variability regions among the alignments. Alignments with equalized length were read and processed in R v4.2.2 [24], running under Rstudio [25], using tools implemented in the packages Biostrings [26] and BiocGenerics [27]. Further, package bio3d [28] was used to calculate Shannon entropy along all alignments, and sequence logos were generated using the package ggseqlogo [29]. All graphs were further constructed utilizing the ggplot2 package [30], and statistical analyses were performed using tools in the Rstatix package [31].

### 2.2. Peptide Selection and Synthesis

Data from in silico analyses were used to design a small library of 15-mer peptides, overlapping by five amino acids, containing sequences encompassing nearly full-length ASP. Peptide sequences reflect subtype-specific signatures and amino acid variations with frequencies above 20% in B, C, and F1 Brazilian alignments. In addition, peptides from HIV gp120 V3 (KRIHIGPGRAFYTTK) and influenza A (YPGEFADYEELREQL) were included as positive controls. A full description of designed peptides, detailing variable positions and relative frequency of amino acid residues among B, C, and F1 world and Brazilian included in our dataset, is available in supplementary Table S3.

Designed peptides were synthesized in cellulose membrane with AutoSpot ASP222 (Intavis, Tübingen, Germany), as previously described [32,33]. Briefly, the Fmoc strategy was used and the synthesis programming was performed using the Multipep v1 software (Intavis, Tübingen, Germany), and coupling reactions were followed by acetylation of residues with acetic anhydride (4%, *v*/*v*) in N, N-dimethylformamide and removal of Fmoc protection groups through the addition of 20% piperidine and carried out sequentially until the whole peptide was generated. After adding the last amino acid from a sequence, lateral chain protective groups were removed with trifluoroacetic acid, tri-isopropyl silane, and water, followed by membrane washing with dichloromethane, water, and ethanol.

### 2.3. Immunoscreening of Peptide Array

Peptide arrays were synthesized to assess IgG and IgM antibody reactivity against pooled plasma samples. For IgM, pooled plasma included equivalent volumes of seven samples from people living with HIV (PLWH) diagnosed during Fiebig phases III to IV and five samples from HIV viremic controllers (individuals presenting low detectable viremia during follow-up in the absence of treatment). The pool used for IgG testing included equivalent volumes of seven samples from PLWH diagnosed during Fiebig V and VI and five samples from HIV Elite Controllers (individuals with undetectable viremia during follow-up, in the absence of treatment). 

Immunoscreenings of the pools were performed as previously described [34]. Briefly, cellulose membranes containing synthesized peptides were washed three times for 10 min with T-TBS pH 7.2 (50 mM Tris, 0.05% Tween, 136 mM NaCl, and 2 mM KCl), followed by blocking of the free sites with T-TBS/1.5% BSA for 10 h at 8 °C. After a new round of washes with T-TBS, each membrane was incubated for 10 h with the sample pool diluted in T-TBS/0.75% BSA in a ratio of 1:150. Next, membranes were rewashed with T-TBS and incubated with antibodies Goat anti-Human IgM (u) alkaline phosphatase (KPL Seracare, Milford, MA, USA) or Goat anti-Human IgG (H + L) alkaline phosphatase (Thermo Scientific, Rockford, IL, USA) for 60 min, followed by washes and incubation with Tropix^®^-CDP-star T2218 (Applied Biosystems, Waltham, MA, USA).

Stained membranes were scanned by the Odyssey FC imager (LI-COR Bioscience, Lincoln, NE, USA), and signal intensities for each spot were quantified with TotalLab TL100 v2009 Software (Nonlinear Dynamics, Durham, NC, USA), using algorithms that compared the intensity between background, spot area, and negative control to characterize spot signal intensity. The spot with the strongest signal for each membrane was reported as having 100% intensity, and all other spots had their intensity values expressed as a relative percentage to this intensity (IR, reactivity index).

## 3. Results

### 3.1. Phylogenetic Analysis of ASP HIV-1 Subtypes

**Figure 1:**
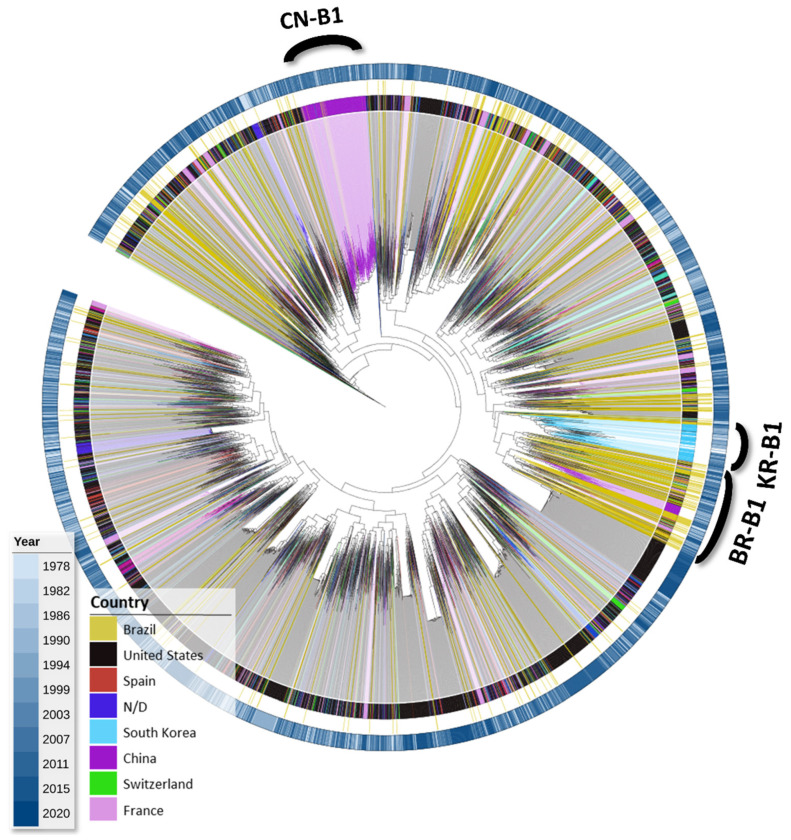
Maximum likelihood tree for worldwide ASP HIV-1 subtype B sequences. The tree was generated from 5771 subtype B sequences in the PerPatient dataset with the Fasttree2 algorithm and plotted/annotated with iTol. The tree was rooted with a subtype D reference clade. Branches are colored, and clades are shaded according to the sequence’s isolation country, as depicted in the legend. The colored concentric ring external to the tree represents the year of the sample collection, which is also colored according to the legend.

Initially, we assessed the phylogenetic relationship among ASP sequences to validate HIV-1 subtype classification and to explore potential variability patterns linked to the geographic origin of each subtype. Due to limits in computational power, maximum likelihood trees for each subtype were generated from PerPatient containing worldwide sequences. For HIV-1 subtype B sequences (Figure 1), two country-related monophyletic clades were identified, one grouping 79% of the Chinese sequences (CN-B1) and another grouping 88% of the South Korean sequences (KR-B1). Brazilian sequences were distributed along the tree; however, a major cluster named BR-B1 was identified, and 20% of all subtype B Brazilian sequences were contained there.

**Figure 2:**
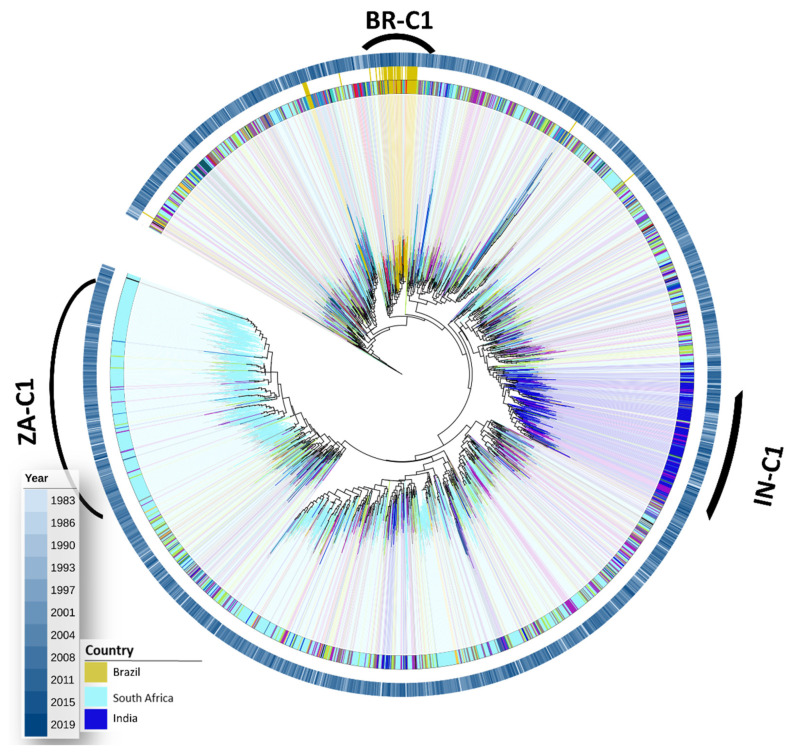
Maximum likelihood tree for worldwide ASP HIV-1 subtype C sequences. The tree was generated from 2119 subtype C sequences in the PerPatient dataset with the Fasttree2 algorithm and plotted/annotated with iTol. The tree was rooted with a subtype D reference clade. Branches are colored, and clades are shaded according to the sequence’s isolation country, as depicted in the legend. The colored concentric ring external to the tree represents the year of the sample collection, which is also colored according to the legend.

The maximum likelihood tree of HIV-1 subtype C (Figure 2) shows that 46% of available subtype C sequences originated from South Africa, and 30% formed a single monophyletic clade identified as ZA-C1. A very representative clade containing 75% of the sequences from India was also observed (IN-C1). Most (80%) of all Brazilian sequences were grouped in a single monophyletic clade named BR-C1.

**Figure 3:**
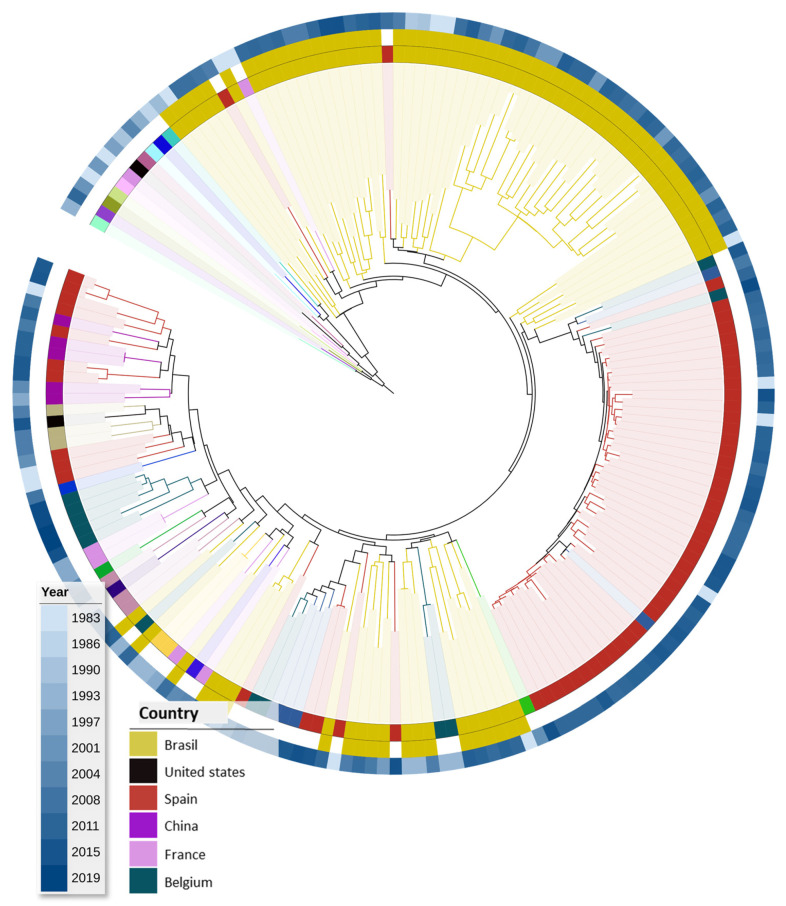
Maximum likelihood tree for worldwide ASP HIV-1 sub-subtype F1 sequences. The tree was generated from 189 sub-subtype F1 sequences in the PerPatient dataset with the Fasttree2 algorithm and plotted/annotated with iTol. The tree was rooted with a subtype D reference clade. Branches are colored, and clades are shaded according to the sequence’s isolation country, as depicted in the legend. The colored concentric ring external to the tree represents the year of the sample collection, which is also colored according to the legend.

Ultimately, most F1 ASP sequences obtained from the LAHSD originated from Brazil or Spain (Figure 3). Within the Brazilian sequences, multiple clusters were verified; however, a predominant clade was not identified.

### 3.2. Genetic Variability of ASP

**Figure 4:**
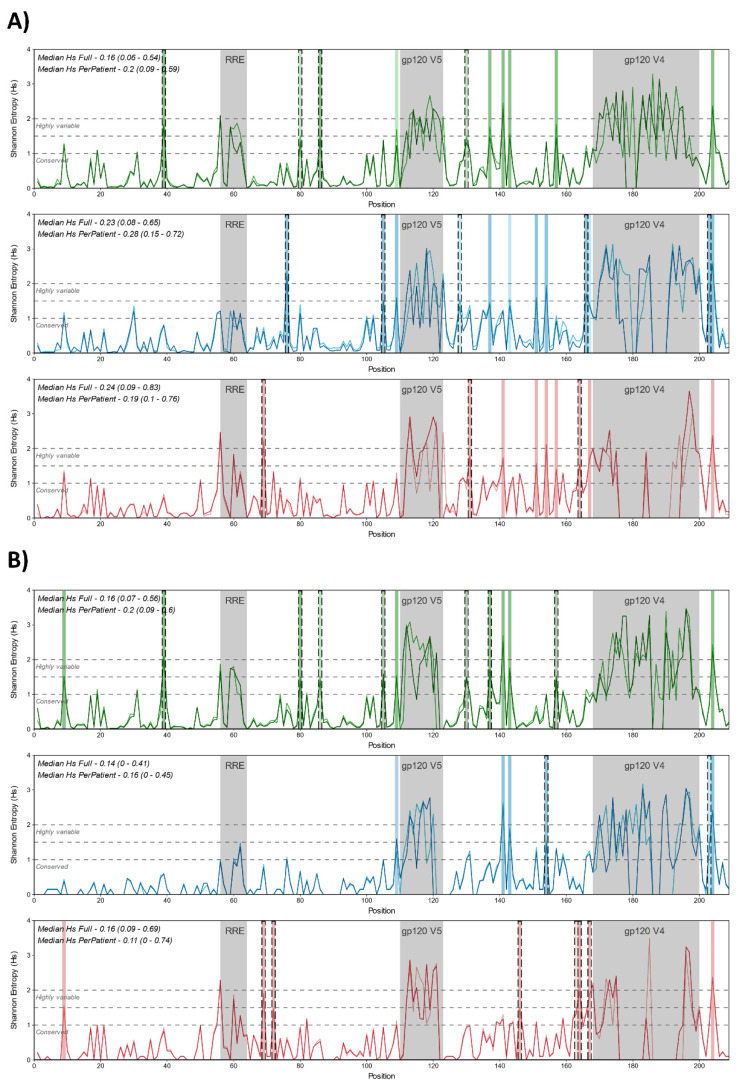
Shannon entropy (Hs) of ASP from worldwide and Brazilian HIV-1 B/C/F1 sequences. The graphs represent the Hs values for each position along the B/C/F1 ASP on alignments containing worldwide (**A**) and Brazilian sequences (**B**). Lines on the graph are colored according to the subtype (green for B, blue for C, and red for F1) and to the dataset used in the alignments (darker tones for the Full dataset and lighter tones for the PerPatient dataset). Along the graph, positions related to hypervariable regions were shaded in gray, and positions with Hs > 1.4 are shaded in colors. Dashed lines indicated positions with Hs > 1.4 and were not shared between the subtypes.

To characterize the genetic variability of ASP and identify diversity patterns related to B, C, and F1 subtypes, we used the amino acid sequence alignments to calculate the Shannon entropy for each position alignment and to identify the variability of each site along the gene. Considering the whole alignment of the full datasets containing worldwide sequences (Figure 4A), the median entropy was low for all subtypes (0.16 [0.06–0.54] for B; 0.23 [0.08–0.65] for C; 0.24 [0.09–0.83] for F1) and did not significantly differ between subtypes. No significant difference was observed when comparing the entropy or graphics between Full/Per Patient analyses, indicating that both datasets were equally representative of the variability along the gene. Considering the Shannon entropy (Hs) threshold of 1.4 worldwide, subtype C sequences presented more highly variable sites than B and F1 sequences (12 vs. 10 vs. 9, respectively) when considering only positions outside the hypervariable regions (V1, V2, and V3). Although at least two subtypes shared the most identified sites, some were exclusively for one of the subtypes (5/12 for C, 4/10 for B, 3/9 for F1) (Figure 4A).

The median entropy of whole alignment for Brazilian sequences was also low for all subtypes (0.16 [0.07–0.56] for B subtype; 0.14 [0–0.41] for C subtype; 0.16 [0.09–0.69] for F1 sub-subtype) (Figure 4B). As for worldwide sequences, no significant differences were observed between subtypes or for Full/PerPatient pairs, but a significantly lower entropy was observed for Brazilian subtype C alignment in comparison to subtype C world alignments (*p* = 0.04 for comparison between Full alignments; *p* = 0.0006 for comparison between PerPatient alignments).

The inspection for highly variable sites depicting entropy values (Hs > 1.4) in Brazilian subtypes B, C, and sub-subtype F1 alignments indicated the existence of 12, 6, and 8 sites outside the variable regions V1, V2, and V3, respectively. Most of these sites observed were exclusively for each subtype: 7/12 for B, 6/8 for 2/6 for subtype C. All sites identified as highly variable among Brazilian alignments also presented high entropy values among worldwide alignments, although some presented values below the 1.4 threshold.

**Figure 5:**
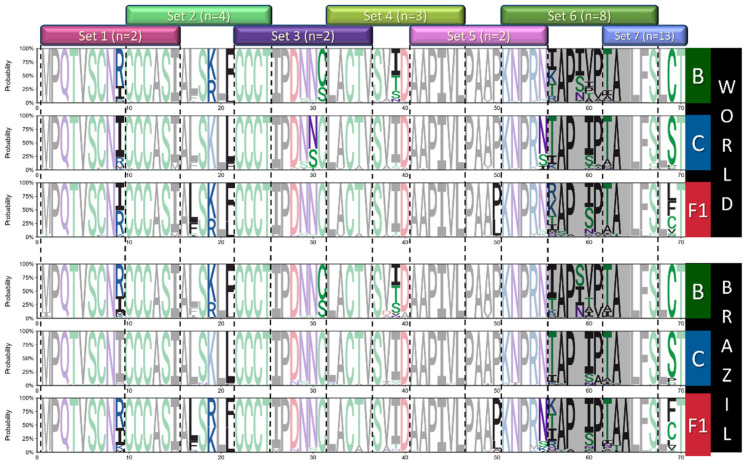
Logos analysis for positions 1–70 of ASP from worldwide and Brazilian B/C/F sequences. Letters indicate amino acid acronyms according to the IUPAC code, and their heights correlate to the frequency of the amino acid in the respective position. Amino acids are colored according to their chemical properties (green—polar; purple—neutral; basic—blue; acid—red; hydrophobic—black), and conserved residues (variability < 20%) are presented in soft tones. The hypervariable region equivalent to ENV RRE was shaded in gray.

**Figure 6:**
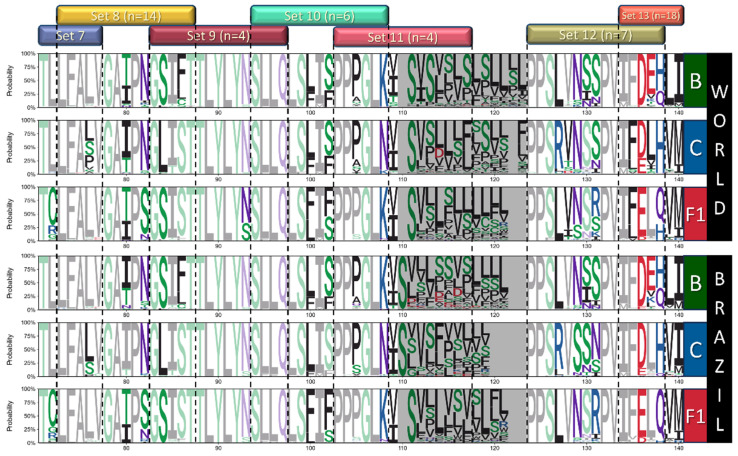
Logos analysis for positions 71–140 of ASP from worldwide and Brazilian B/C/F sequences. Letters indicate amino acid acronyms according to the IUPAC code, and their heights correlate to the frequency of the amino acid in the respective position. Amino acids are colored according to their chemical properties (green—polar; purple—neutral; basic—blue; acid—red; hydrophobic—black), and conserved residues (variability < 20%) are presented in soft tones. The hypervariable region equivalent to gp120 V5 was shaded gray.

**Figure 7:**
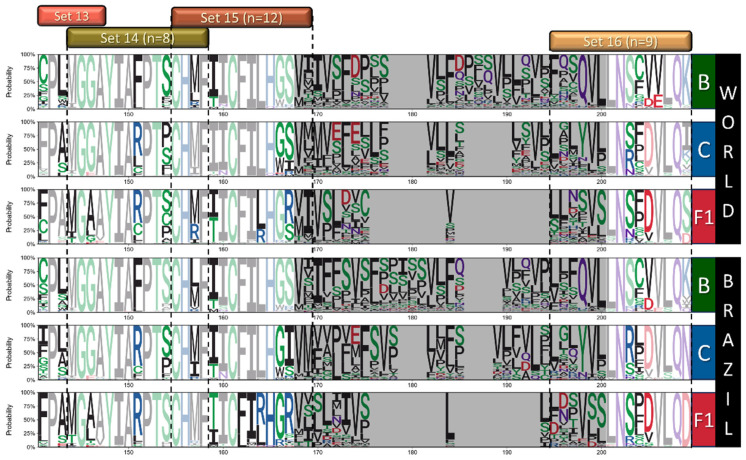
Logos analysis logos for positions 141–209 of ASP from worldwide and Brazilian B/C/F sequences. Letters indicate amino acid acronyms according to the IUPAC code, and their heights correlate to the frequency of the amino acid in the respective position. Amino acids are colored according to their chemical properties (green—polar; purple—neutral; basic—blue; acid—red; hydrophobic—black), and conserved residues (variability < 20%) are presented in soft tones. The hypervariable region equivalent to gp120 V5 was shaded gray.

Following entropy analysis, we used Full datasets to generate and analyze sequence logos for each alignment to characterize the amino acid variations among sites with higher entropy and to identify subtype-specific signatures among regions with lower entropy. Logos are presented in three parts to cover all ASP regions (Figure 5, Figure 6 and Figure 7), and positions presenting amino acid frequencies superior to 20% are highlighted. Based on this analysis, we were able to characterize relevant variability in 23 sites from subtypes B and C and 30 sites from F1 worldwide alignments. Among Brazilian alignments, 22, 17, and 29 variable sites were identified for B, C, and F1, respectively. In addition, we identified several conserved sites that presented subtype-specific signatures based on worldwide (11 for B; 10 for C; 6 for F1) and Brazilian alignments (12 for B and C; 10 for F1).

Summarizing and discarding regions with low alignment accuracy, several sites with higher variability (H_s_ >1.5) were identified among the datasets (25 worldwide, 20 Brazilian). Among sites with H_s_ < 1.4, sequence logos allowed the identification of 23 other sites with subtype-specific signatures. Altogether, amino acid variations with frequencies > 20% in the 48 variable sites identified were included in 92 peptides, divided into 15 sets, representing near full-length ASP. The relative positions of the sets and the full peptide list are available in Figure 5, Figure 6 and Figure 7 and Supplementary Table S3.

### 3.3. IgM and IgG Responses against ASP

**Figure 8:**
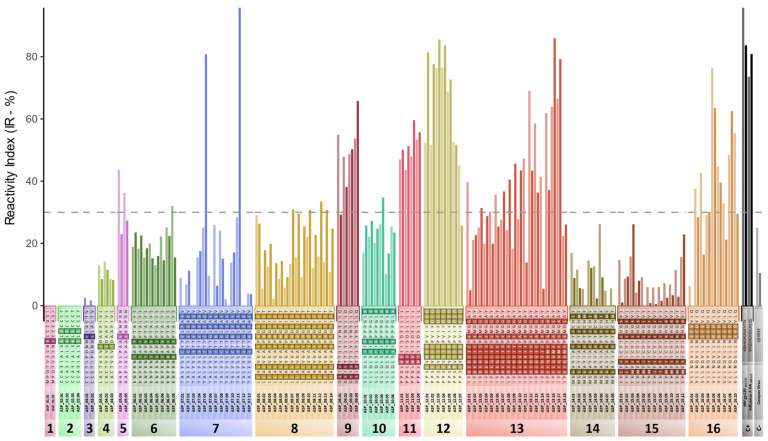
IgG and IgM responses against ASP peptides. The graphic shows IgG and IgM reactivity indexes (IR) obtained for each peptide. Labels in the x-axis contain the ID of the peptide and its sequence, with residues containing intra-set variations highlighted. Results from the positive controls, gp120 V3 and Influenza A antigens, and from the negative control are displayed in the last two sets. Results for each peptide are shown in pairs, with lighter-toned bars representing IgM IR and darker-toned bars representing IgG results. The dashed horizontal line indicates the 30% threshold.

The designed peptides were synthesized in cellulose arrays and employed to assess the humoral response against ASP. The reactivity against IgG and IgM was analyzed in samples of PLWH with recent HIV infection and HIV controllers. Figure 8 presents the responses for each peptide set.

Absent or weak responses were observed against the N-terminus of ASP, covering peptide sets 1 to 4. Thus, significant immunogenic regions are not observed until the left flank of ASP_V1_; peptide Set 5 exhibited IgM IRs > 20% and IgG IRs > 30%. Considering the obtained immune responses for sets 4 and 6, which partially overlap with Set 5, we infer that the immunogenic core for this region comprises the amino acids from the center and right end of Set 5.

The most robust responses against ASP, for both IgG and IgM, are observable in the middle section of the protein. Regarding IgG, Set 9 presented IRs > 47% for all tested peptides, displaying increased reactivity compared with sets 8 and 10. Therefore, this may indicate that the central region of Set 9 is another immunogenic core. A different pattern was observed for IgM, as the amino acid variations presented at the initial portion of the peptides appeared to affect the reactivity, with IRs > 50% observed only for the peptides containing the motifs GLIS and GSIS, which are more frequent in sequences from subtypes C and F1. The IgM IRs nearing 30% for several peptides from Set 8, in contrast to lower values for IgG, indicate that the regions targeted by IgG and IgM are distinct. Response to IgG targets the central region of Set 9, while IgM targets the region between sets 8 and 9.

Stronger immunogenic responses were also observed for sets 11 and 12, which flank the ASP_V2_ region. For Set 11, a possible immunogenic core related to this set was identified in the immediate left region that flanks the hypervariable region ASP_V2_. Lower reactivities for peptides in Set 10 in comparison to Set 11 corroborate this hypothesis. For Set 12, the mean immune response detected for the seven tested peptides was 60.5% for IgG and 68% for IgM. The strongest response was verified against peptides that presented an isoleucine at position 130 (peptides 12.3 and 12.4), which was detected in about 40% of the dataset obtained from HIV-1 subtype B Brazilian sequences. 

Furthermore, sets 13 and 16 presented robust responses for some peptides, although each set had a high level of variability. For Set 13, IRs varied between 18.3 and 69.0 for IgG and 8.0 and 86.0 for IgM. The lowest response was observed among peptides 13.09 to 13.14, which contain motifs more prevalent in subtype C sequences, and among peptides 13.15 to 13.18, which include motifs characteristic of sub-subtype F1. In Set 16, IRs ranged from 6.27 to 76.0, and the lowest responses were observed against a peptide that presented a valine at position 205. Notably, valine is the more prevalent amino acid at this position for subtype B but not for C and F1.

## 4. Discussion

Since the discovery of HIV, most of its genes and correlated proteins have undergone extensive characterization, revealing their functions, insights into the replication cycle, patterns of viral evolution, and their involvement in the immunological response. Despite that, data regarding the characteristics of ASP are mostly scarce and preliminary, even though the first description of the gene dates to the end of the 1980s [6] and recent data demonstrated that ASP protein is associated with the viral envelope [16]. Considering this, our work aimed to investigate the immunogenic potential of ASP by assessing IgM and IgG response magnitude throughout the whole protein extension, considering the amino acid signatures and the most prevalent mutations for HIV-1 subtypes B, C, and F1, the most pervasive HIV-1 subtypes in Brazil. 

By using pools of plasma samples obtained from untreated PLWH in the initial phases of infection and HIV controllers, we could detect high IgM and IgG responses against several ASP regions, mainly in the central area of the protein. Along with the data recently published by Savoret et al. [18], our study corroborates the results obtained in 1995 by Vanhée-Brossollet et al. [17], which identified anti-ASP antibodies in the sera of PLWH and first hinted at the potential of ASP as a target of the immune response. Since then, there has been scarce data evaluating ASP immunogenicity in the literature, with three other studies evaluating cellular responses [4,5,35], and the current research is the third describing patterns of humoral response against ASP. Despite its unknown function, identifying ASP as a transmembrane envelope protein [16] strongly indicates its potential as a surface viral antigen and viable candidate for vaccine targets alongside gp120, yet it has not been thoroughly explored. Considering recent failures in clinical trials evaluating vaccine candidates using mosaic antigens [36], the improved knowledge about ASP would help understand whether ASP could be utilized in immunizations and may significantly contribute to developing and designing future vaccine clinical trials. 

The viral genetic diversity greatly impacts the effectiveness of anti-HIV immune responses, leading to immune escape during the chronic phase of infection [37,38,39,40], which is a challenge to developing an effective preventive vaccine [41]. The env gene is the most variable of the virus genes, presenting a variability that can reach up to 30% between isolates from different subtypes and is primarily accumulated in the gp120′s hypervariable regions [42,43]. All this variability could directly impact ASP and should affect the conservation of epitopes targeted by the immune response, as the antisense gene overlaps the *env* region that includes regions V4/V5 hypervariable loops of gp20. Hence, evaluating the impact of ASP diversity alongside immunogenicity studies is crucial for selecting the best antigen candidate.

In the present study, we focused the diversity analyses on viruses from subtypes B, C, and sub-subtype F1, which were the most prevalent HIV subtypes in Brazil [44]. Phylogenetic analyses performed with our datasets revealed only a few clade grouping sequences mainly isolated from a single country, including a Korean and a Chinese clade for subtype B and two clades from India and Zaire for subtype C. A main subtype B and C sequence clade was observed for Brazilian sequences. Still, the distribution of the Brazilian sequence along the maximum likelihood trees points out the genetic variability of ASP with low geographic structuration. 

Regarding genetic variability, Shannon entropy analyses revealed a general low entropy for the ASP gene outside the hypervariable regions, which did not diverge among the subtypes evaluated. Differences between worldwide and Brazilian datasets were observed when comparing subtype C alignments, which reflects the higher degree of similarity between Brazilian sequences compared with sequences from other countries. In the case of sub-subtype F1, entropy data was comparable between Brazilian and worldwide datasets. Therefore, it suggests that variability in Brazilian sequences is equivalent to the ASP diversity observed globally.

Although the most variable sites identified during entropy analyses are shared by different subtypes, about 40% of the highly variable sites identified for each subtype were conserved among the other two evaluated subtypes, indicating a diversity pattern that should be subtype-specific. In addition, several subtype-specific variations were identified in sites depicting low entropy throughout Logo data. Therefore, it was incorporated into peptide design, ensuring a spectrum of peptides covering subtype-specific signatures and comprising most of the variability that could impact immune response. This design reflects differences in the reaction magnitude driven by different subtypes.

Immunoscreening results showed that the N-terminal region of ASP is poorly immunogenic, with barely any response detectable against the first 35 amino acids of the protein. Similar data was demonstrated by Savoret et al., in which the deletion of the first 26 amino acids from ASP did not result in weaker responses compared with experiments performed with full-length ASP [18]. Although this initial portion of the protein is characterized by the presence of two cysteine triplets, a motif associated with the binding of metallic ions at N-terminal regions [45], Cassan et al. demonstrated that most viruses from subtype A and its recombinants presented a stop codon located at position 12 and an alternative start codon at position 29 [12]. Thus, these results suggest that this region is optional for protein functionality, given the high prevalence of this subtype and its recombinants.

Despite lower-magnitude responses along the first third of the protein, IgG responses reached nearly 40% for peptides from Set 5. Although those responses were lower than observed for the center of the protein, it deserves to be highlighted, as this region includes a conserved double proline-rich motif (PxxPxxP), and it is located immediately before the portion equivalent to the gp120′s RRE domain. This region was also part of one of the peptides previously tested by Vanhée-Brossollet et al. [17], and it was highlighted by Savoret et al. as a target region of antibodies against ASP [46]. In addition, we also observed a slight difference in the response between the two tested peptides from Set 5, with a reactivity of about 10% lower against the peptide presenting the mutation. P50L, which was detected in about 30% of the subtype B sequences, affects the central proline of the proline-rich domain and indicates lower immunogenicity for this region when the motif is not present.

The stronger responses observed in our study were detected for peptides targeting the protein’s central region, which is presumed to compose the ASP’s ectodomain [14] and agrees with two previous studies [17,18]. Savoret et al. indicated the presence of epitopes in the region delimited by sets 7–13 in our research, while our peptides from Set 12 comprise the region targeted by one of the peptides tested by Vanhée-Brossollet et al. In addition to these data, we identified that the main epitopes involved in the immunogenicity of the ASP central region are located in the sites covered by sets 9, 11, and 12.

For Set 9, we observed differences in the region targeted by IgM and IgG, with a possible displacement of the targeted epitopes between the two classes of antibodies. In this case, IgG responses were similar between all peptides, but IgM responses against peptides presenting characteristic motifs from subtypes C and F1 were about 40% higher than those from subtype B. Besides the influence of viral diversity in the pattern of immune response observed, this result also indicates that both IgG and IgM target different epitopes in the region covered by Set 9, with the IgG epitope located in a more conserved region and, thus, resulting in a good candidate for vaccine target.

Besides the Set 9 region, the region equivalent to gp120 V5 (sets 11 and 12) should be interested in immunogenicity, as the two sets directly flanking the region presented strong responses. The participation of hypervariable loops from gp120 in the immune response was extensively studied. The V3 region contains one of the most frequently recognized HIV epitopes recognized by antibodies; however, analyses of the results from the RV144 clinical trial indicated that vaccine efficacy correlated positively with the magnitude of anti-V1 and anti-V2 antibodies [47,48]. While the sequence diversity in the peptides from Set 11 did not appear to affect both IgG and IgM responses, Set 12 presented different patterns that could be related to both Ig class and viral sequence. For the last, the IgM response was consistently high for all variants commonly found in subtype B but lower for peptides with mutations related to subtypes C and F1.

In contrast, IgG responses were lower only for peptides containing motifs related to subtype F1 and stronger when isoleucine was present in position 130. These results indicate that Set 12 epitopes were highly immunogenic for subtypes B and C but not F1. The sequence’s high diversity should have a greater impact on the response magnitude.

Sets 13 and 16 also presented stronger responses for some peptides. Still, the higher degree of variation in the magnitude of response observed between the peptides indicated that the region is not an immunogen with a broad range. The divergent results observed for Set 16, which represented ASP’s C-terminal region, plus the low responses observed for sets 14 and 15 related to the observation from Savoret et al. that antibodies did not target the C-terminus of the protein. However, the lack of response observed in their study was probably impacted by the sequence used in their antigen, which contained the variation present in peptide 16.1, which was barely recognized in our study for both IgG and IgM.

Lastly, the lower reactivities for sets 7–8 and 14–15 could be related to the presence of two putative transmembrane domains previously identified [14]. For these sets, a relevant decrease in the mean response can be observed in comparison to their adjacent sets, indicating that the exposition of these residues to the immune response is mostly limited. In addition, responses against peptides that represent the N-terminal and C-terminal regions of ASP, which are presumed to constitute intracellular portions of the protein, mostly reached levels below the IR threshold (30%), which is compatible with a low exposition feature.

Although the present work provides a comprehensive overview of the response to anti-ASP antibodies, it is important to note that the methodology employed has some limitations. First, the use of pooled samples could bias the reactivity data, and lower responses observed for some peptides containing subtype-specific variations could result from the fact that most of the samples from the pool were derived from individuals not infected by viruses from that specific subtype. Despite that, the samples included in our pools were mostly derived from individuals infected with HIV viruses from subtype B, which is the most prevalent in Brazil [44]. Thus, the observed increased reactivities for peptides containing motifs or signatures characteristic of subtypes C and F1 when compared with peptides containing variations associated with subtype B show that the employed methodology could provide some resolution for these evaluations despite a probable bias. Similar bias could affect comparisons between IgM and IgG data since different pools were used to assess reactivities against each class, and an unbiased analysis would require the testing of longitudinal samples obtained from a single individual. Despite that, spot synthesis followed by immunoblot is a well-standardized technique that allows a great throughput for broad screening and whole protein mapping, allowing the identification of general immunogenicity patterns. More specific questions and confirmations of those results should be further explored by testing individual samples obtained from PLWH in different clinical settings and infected with viruses from different subtypes, allowing for confirmation of clade-specific antigenic determinants and identification of immune response patterns associated with stage of infection and/or treatment administration, as previously shown [46].

In addition, we could not identify and verify the response to conformational epitopes since the evaluated immune response was directed against linear peptides. Despite that, our results mostly agreed with the results obtained by Savoret et al., which used methodologies that allowed properly folded antigens. Also, the absence of a model containing the tertiary structure of ASP and a lack of knowledge regarding its native conformation in vivo pose challenges to precisely identifying the location of each epitope and determining whether they are accessible to antibody binding. Despite these obstacles, identifying antibodies in biological samples serves as evidence that the immune response of the individuals had indeed targeted the tested peptides.

## 5. Conclusions

In summary, our results provide a more refined map delineating the immunogenic regions of ASP and show that IgM and IgG responses are preferentially directed towards epitopes at the middle region and the C-terminus of ASP. In addition to identifying those regions, the use of data regarding variability patterns that characterize subtype-specific signatures for B, C, and F1 HIV variants allowed us to determine the effects of mutations in the IgG and IgM reactivities and the presence of sets with low IR variance, indicating regions with antibody cross-reactivity between HIV from B, C, and F1 subtypes.

## Data Availability

Data supporting this manuscript may be available upon reasonable request to the corresponding author.

## Supplementary Materials

The following supporting information can be downloaded at: https://www.mdpi.com/article/10.3390/vaccines12070771/s1. Supplementary Table S1. List of HIV ASP sequences retrieved from LAHSD and included in analyses datasets; Supplementary Table S2. ASP sequences were obtained from the Los Alamos HIV sequence database and stratified according to geographic information and genetic classification; Supplementary Table S3. Statistics of residue frequencies among B, C, and F1 subtypes datasets for each evaluated peptide; Supplementary Figure S1. Median IgG and IgM responses against ASP peptides. The boxplots represent median and IQR for IgG and IgM reactivity indexes (IR) obtained for each peptide sets. Results are shown in pairs, with lighter tone bars representing IgM IR and darker tone bars representing IgG results. The dashed horizontal line indicates the 30% threshold.
