# Supplementary material for: Patterns of Diversity and Humoral Immunogenicity for HIV-1 Antisense Protein (ASP)
Source: Vaccines (Basel). 2024 Jul 13;12(7):771. doi: 10.3390/vaccines12070771 (PMC11281420; doi:10.3390/vaccines12070771)
Supplement: Supplementary file 1 [file vaccines-12-00771-s001.zip › Supplementary Figure S1.pdf]

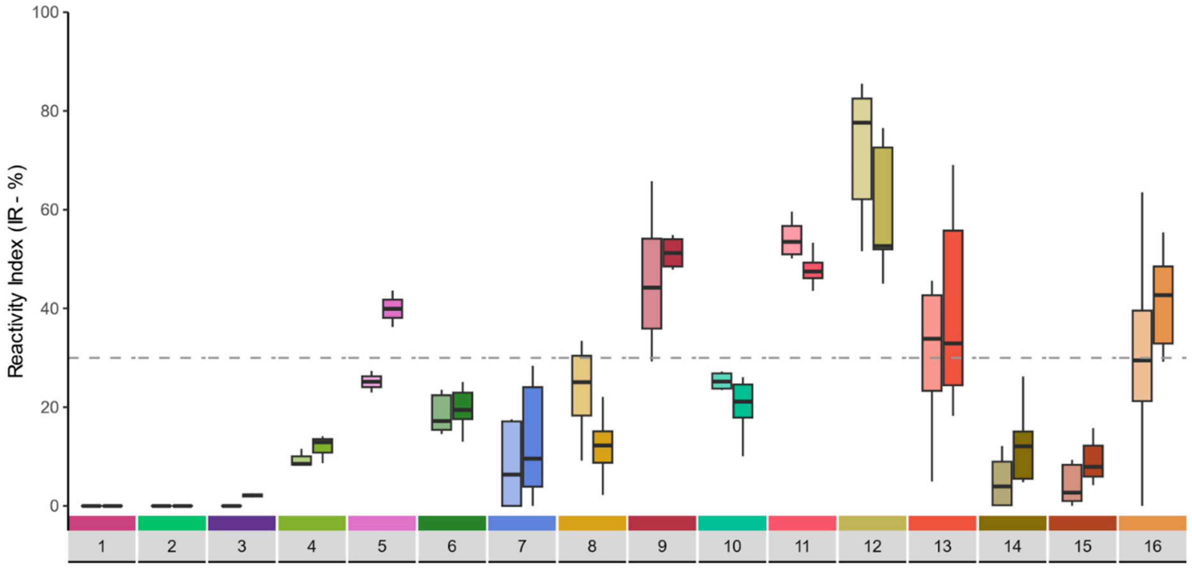


**Supplementary Figure S1. Median IgG and IgM responses against ASP peptides.** The boxplots represent median and IQR for IgG and IgM reactivity indexes (IR) obtained for each peptide sets. Results are shown in pairs, with lighter tone bars representing IgM IR and darker tone bars representing IgG results. The dashed horizontal line indicates the 30% threshold.
